# Multiple Sclerosis and Microbiome

**DOI:** 10.3390/biom12030433

**Published:** 2022-03-11

**Authors:** Jana Lizrova Preiningerova, Zuzana Jiraskova Zakostelska, Adhish Srinivasan, Veronika Ticha, Ivana Kovarova, Pavlina Kleinova, Helena Tlaskalova-Hogenova, Eva Kubala Havrdova

**Affiliations:** 1 Department of Neurology and Centre of Clinical Neuroscience, First Medical Faculty, Charles University and General Medical Hospital in Prague, 12800 Prague, Czech Republic; adhish25@gmail.com (A.S.); veronika.ticha@vfn.cz (V.T.); ivana.kovarova@vfn.cz (I.K.); pavlina.kleinova@vfn.cz (P.K.); eva.kubalahavrdova@vfn.cz (E.K.H.); 2 Laboratory of Cellular and Molecular Immunology, Institute of Microbiology of the Czech Academy of Sciences, 14220 Prague, Czech Republic; zakostelska@biomed.cas.cz (Z.J.Z.); tlaskalo@biomed.cas.cz (H.T.-H.)

**Keywords:** multiple sclerosis, microbiome, gut-brain axis, disease-modifying drugs

## Abstract

The composition of microbiota and the gut-brain axis is increasingly considered a factor in the development of various pathological conditions. The etiology of multiple sclerosis (MS), a chronic autoimmune disease affecting the CNS, is complex and interactions within the gut-brain axis may be relevant in the development and the course of MS. In this article, we focus on the relationship between gut microbiota and the pathophysiology of MS. We review the contribution of germ-free mouse studies to our understanding of MS pathology and its implications for treatment strategies to modulate the microbiome in MS. This summary highlights the need for a better understanding of the role of the microbiota in patients’ responses to disease-modifying drugs in MS and disease activity overall.

## 1. Introduction

Multiple sclerosis (MS) is a chronic autoimmune inflammatory disease of the central nervous system (CNS). The disease leads to the formation of inflammatory lesions in the CNS, in which myelin sheaths break down and demyelinated axons are damaged. This leads to neurological symptoms such as impairment of motor functions, sensitivity, balance, vision, and sphincters, but also fatigue, depression, and cognitive changes.

This disease has a complex etiology with genetic and environmental factors playing a significant role. Studies in identical twins show that the genetic influence on the disease risk is only about 30% [1] with human leukocyte antigen (HLA) genes being the most important factor [2]. Environmental factors such as EB virus infection [3,4], vitamin D deficiency (increased prevalence with distance from the equator), smoking (apparently as an epigenetic factor), obesity, stress, and other unknown factors were suggested as being involved in the disease [5]. Current evidence supports the microbiota as a factor contributing to inflammation and autoimmune reactions. Large-scale projects such as the Human Microbiome Project and the Metagenomics of the Human Intestinal Tract (MetaHIT) have been involved in the establishment of the link between the gut microbiota and human health and disease [6,7,8]. 

Over the past decade, there has been a growing recognition of the role of microbiota in the regulation of the so-called gut-brain axis in the maintenance of homeostasis. The gut-brain axis as a bidirectional signaling communication network is involved in the regulation of neural, hormonal, and immunological pathways and its dysfunction is implicated in various inflammatory, psychiatric, and neurodegenerative disorders (with MS being one of them).

## 2. Microbiota and Human Health

Microorganisms that colonize the human gastrointestinal tract (GIT) are symbiotic under steady state conditions and contribute to the homeostasis of the human host. The term microbiome refers to the collective genetic material contained in these microorganisms. Interestingly, even though there is a remarkable variation in the diversity and abundance in the microbiota that occupy different habitats, metabolic pathways remain stable within a healthy population [6]. 

The composition and diversity of the microbiota vary greatly according to their location in the GIT and also vary in composition according to the age of the individual [9]. Several factors such as diet, mode of delivery (vaginal or caesarean), use of antibiotics, geography, and environmental exposure influence their intraindividual variation and development [10]. The recent emergence of evidence from animal studies (using probiotics, antibiotic treatments, and germ-free (GF) mouse models) shows the importance of commensal microbes in normal physiological functioning and suggests that microbes influence postnatal and adolescent development [11,12]. 

The microbiota also plays a fundamental role in the functioning of host innate and adaptive immune responses. There is evidence that suggests that the human immune system modulates itself in order to maintain the balance between symbiotic, commensal, and pathological microorganisms. This relationship between the host immune system and microbiota induces protective mechanisms against pathogens and is involved in the regulatory response towards the tolerance of harmless antigens. 

## 3. Microbiota and Its Role in the Immune System

Alterations of the host microbiota due to changes in diet and lifestyle, antibiotic overuse, and the eradication of helminth nematodes, especially in developed countries, may have predisposed individuals to flawed microbiota in terms of their immune response [13]. This phenomenon could partially account for the dramatic rise in inflammatory and autoimmune disorders, especially in Western society, where the greatest disbalance in the symbiotic relationship between the host and the gut microbiota exists [12,14].

Conclusions of studies of microbiome profiling, using methods such as 16S rRNA and metagenomic shotgun sequencing technologies, suggest that changes in the relative composition of intestinal microbiota, known as intestinal or gut dysbiosis, play a role in several autoimmune diseases. Evidence suggests that intestinal microbiota modulate the immune system and affect the neural and endocrine systems of the gut [15].

Germ-free mice studies confirm the role of gut microbiota in the healthy functioning and development of the immune system [16]. Commensal microbiota produce a plethora of antigens that act as immune stimulants while also stimulating the production of antimicrobial factors, antibodies, and cytokines, which help to maintain an equilibrium [17]. Regulation of the host-microbiota relationship in the GIT involves effectors such as IL-17-expressing T cells (Th17) as well as regulatory T cells (Treg), as evidenced in groups of Clostridium bacteria IV and XVI and segmented filamentous bacteria. Interestingly, not only live bacteria but also bacterial components can induce Tregs within the GIT [18]. Innate cells such as specific types of dendritic cells stimulated by gut microbiota also play an important role in the induction of Treg cells and the generation of Th17 cells, which link the innate and adaptive immune systems [12].

Toll-like receptors (TLR) and related microbe associated molecular pattern recognition (MAMP) ligands are important for B-cell regulation. Microbial metabolites such as short-chain fatty acids (SCFAs) and adenosine triphosphate (ATP) have shown to be significant for mucosal antibody production. SCFAs exert an anti-inflammatory effect via the inhibition of histone deacetylases (HDACs) on Treg and microglia. This process is mediated by G-protein-coupled receptors (GPCR) primarily expressed by epithelial cells and some myeloid cells. Moreover, SCFAs are integrated into cellular metabolism following their conversion to acetyl-CoA, and this greatly affects the cellular metabolism in T and B cells [19].

A communication network between intestinal epithelial cells (IECs), the gut microbiota, and immune cells regulates inflammation through effectors from the immune system (TLRs) and microbes [lipopolysaccharide (LPS)] and maintains the intricate balance between them. IECs, with their tight junctions, create a barrier between host immune cells and gut microbiota and are important in distinguishing between beneficial commensal bacteria and pathogenic bacteria. This network maintains homeostasis at the mucosal surface. A breach in this barrier causes increased gut permeability and allows for the systemic dissemination of the bacteria and/or their products. This so-called “leaky gut syndrome” is associated with several autoimmune and inflammatory diseases [20].

## 4. Autoimmunity and Multiple Sclerosis

Complex interactions within the gut-brain axis may be relevant to the pathophysiology of neurological disorders, autoimmune disorders, and multiple sclerosis specifically. 

Clinical symptoms of multiple sclerosis (MS) typically appear between the 20th and 30th years of life; however, the disease starts years before as is documented by initial magnetic resonance imaging (MRI) of the brain. The diagnosis of MS is based on a clinical event that leads to a discovery of demyelinating lesions of different ages in the brain and/or spinal cord through MRI (dissemination in time and space) and evidence of immune activation specific to the cerebrospinal fluid (CSF) compartment (i.e., the presence of oligoclonal bands in CSF, and an increased IgG index) [21].

The crucial role of activated myelin antigen-specific T cells in the pathogenesis of MS has been evidenced by experimental autoimmune encephalomyelitis (EAE), the animal model of MS [22]. The cause of the activation of these auto-aggressive clones, which most likely occurs in the deep cervical lymph nodes, remains unclear. Recurrent infections, molecular mimicry, and the influence of pro-inflammatory microbiome products have been considered as potential triggers [23]. Histopathological studies demonstrate that activated auto-aggressive T cells cross the blood–brain barrier and form foci of inflammation in the vicinity of small venules [24]. This environment, rich in pro-inflammatory cytokines and toxic substances (such as free radicals), induces myelin and axonal damage. Axonal loss is the basis of permanent disability in MS and correlates with an increase in brain atrophy on MRI [25]. Increased levels of serum neurofilaments reflect axonal damage in MS and serve as an important biomarker of disease activity [26]. 

Our understanding of the pathophysiology and the development of treatment options for MS has long been influenced by the T cell-dependent EAE model. The efficacy of anti-CD20 treatment has demonstrated the definite role of B cells, which are believed to activate T cells, produce pro-inflammatory cytokines, function as antigen-presenting cells, and produce antibodies. Intrathecally produced antibodies detectable as oligoclonal bands have been used since the 1960s to support the diagnosis of MS. The recent work of Bjornevik et al. highlighted a long-suspected connection between EBV and the development of MS [3]. Multiple hypotheses have been considered to explain the contribution of EBV to the pathophysiology of MS, including the aberrant activation of autoreactive immune cells in the periphery, the ability of EBV to drive a dysregulated pro-inflammatory B cell response, a theory of the cross-activation of T cells after EBV infection, and a hypothesis of coincident expression of aB-crystallin in lymphoid cells and oligodendrocytes [27]. As EBV can persist in memory B cells, it was also hypothesized that EBV harboring B lymphocytes [28] migrate into the CNS, spread, and lead to tissue damage due to their response to the infected cells. It is also known that in the later stages of MS, B lymphocytes form microscopic nodules on meninges, which resemble the nodal environment in germinal centers [29]. 

Acute attacks of MS are treated with high doses of corticosteroids, but the overall prognosis can be changed only by early treatment with disease-modifying drugs that now includes a number of options: interferon beta, glatiramer acetate, teriflunomide, dimethyl fumarate, fingolimod, cladribine, natalizumab, ocrelizumab, alemtuzumab, ofatumumab, siponimod, and non-specific immunosuppressive drugs. It is currently not known what individual characteristics of patients determine the optimal treatment option and whether the microbiome plays a role in the efficacy of treatment in MS. 

## 5. Connection between the Microbiome and Multiple Sclerosis

The influence of the microbiome could explain the unidentified environmental risk factors in the development of MS, and it may be involved in influencing the immune system and the course of the disease. It is now well known that pathological changes in the intestinal microbiota (dysbiosis) can affect inflammation in the CNS [30]. 

In patients with MS, it was found that the composition of their microbiota was relatively different from that of healthy individuals [31]. Most importantly, the diversity of the microbiome was reduced. Studies are not entirely consistent, but they mostly report an increase in Akkermansiaceae and Methanobacteriaceae and a decrease in SCFA production corresponding to a lower presence of Bacteroidetes and Clostridia clusters XIVa and IV [17,32,33]. In the animal model, the alteration of these ratios leads to alleviated manifestations of the disease, probably due to the induction of regulatory T cells. 

The animal model of MS (experimental autoimmune encephalomyelitis (EAE)) is induced in susceptible animals by the adoptive transfer or active induction of autoreactive myelin-specific lymphocytes [22]. After the multiplication of antigen-specific lymphocytes in the regional node, the lymphocytes migrate to the CNS and form inflammatory foci, which lead to symptoms similar to MS. Experiments based on EAE models of MS have given us an insight into the adaptive immune response, demyelination, and axonal injury in MS; however, the EAE models represent only a fraction of MS pathology and are limited in modeling the MS onset in humans. 

The relapsing-remitting model of spontaneously developing experimental autoimmune EAE has been used to demonstrate the involvement of microbiota in disease development [34]. 

The immune system of mice that are kept in a germ-free environment behaves differently from that of mice who are exposed to common microbes found in their environment. The transmission of intestinal microbiota from MS patients leads to the induction of spontaneous EAE in mice. The study of microbiota samples from twins, where one is healthy and the other has MS, reveales that the influence of the genetic background and the environmental differences in childhood development is minimal [31]. The colonization of the gut by microbes of MS patients has led to a far more serious experimental disease [31,34,35].

It has not yet been possible to characterize an accurate description of dysbiosis in MS; as such, the possibility of the therapeutic application of one or more species of bacteria does not look very promising [36]. The differences to the healthy population are not very substantial. In addition, the causality of these findings in humans is not proven. It is speculated that the microbiome may play a role in the susceptibility to MS in a given genetic background, but therapeutic interventions are not yet practically possible to implement.

It is, therefore, necessary to systematically collect a large number of samples from a diverse set of patients (treated vs. untreated, different stages of the disease), and to establish conclusive evidence of correlations between intestinal dysbiosis and MS.

## 6. Hygiene Hypothesis, the Microbiome, and MS

**Figure 1:**
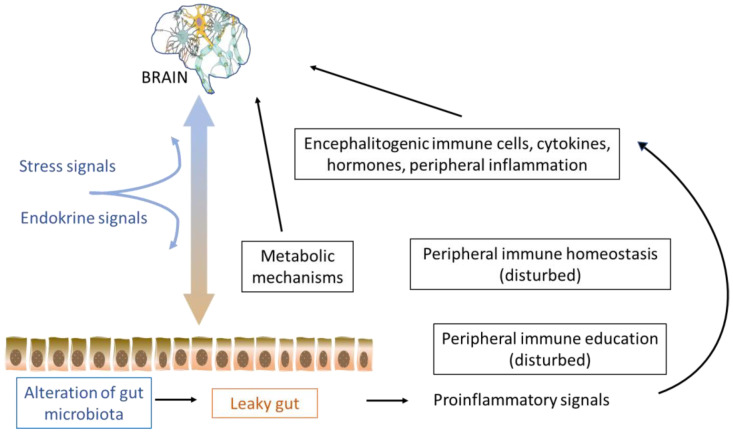
Schematics of the possible interaction between the gut and CNS via the immune system in multiple sclerosis.

The observation of an increase in allergic and autoimmune diseases in industrialized nations in recent decades has led to the development of a “hygienic hypothesis” that claims that this is the result of an environment that is too clean to properly stimulate, nurture, and regulate the immune system. However, it now seems that some revision of this hypothesis in connection with autoimmune diseases is needed [37]. Environmental purity is mostly related to the relative lack of microbes and microbe-associated molecular patterns (MAMPs) [38]. Higher TLR2 expression and a significantly increased response to TLR2 ligands have been observed in MS patients, suggesting a lack of regulation, a mechanism called TLR tolerance, which arises upon the repeated exposure to an adequate TLR2 stimulus. TLR tolerance is a phenomenon that is associated with increased phagocytosis, decreased macrophage antigen presentation, decreased TNF-alpha expression, and increased IL-10 expression. The microbiome is an important part of our organisms and as such can stimulate the immune system as well as induce tolerance. In this way, the microbiome seems to influence the excessive immune response that underlies autoimmune diseases (Figure 1). It could be expected that the modulation of microbiota composition will be implemented in future preventive and therapeutic approaches [39].

As early as 1996, a prerequisite study of the enabling interactions between the intestinal microbiota and the CNS was published, i.e., increased intestinal permeability [40]. The condition allows macromolecules, toxins, commensal, and pathogenic bacteria to enter the bloodstream through the intestinal epithelium. It can be considered a kind of biomarker of both local and distant immune-mediated disorders. In 2014, this increased intestinal permeability was demonstrated even before the onset of symptoms in EAE [41]. An impairment of the intestinal barrier complex (the microbiome, the mucin layer, gastrointestinal secretion, enterocytes, immune cells, gut vascular barrier, and liver barrier) was demonstrated in human studies of the metabolic syndrome and obesity, inflammatory bowel disease, stroke, type I diabetes, systemic lupus erythematodes, and MS [42]. There was also an effort to understand the relationship between the intestinal barrier and the quality of the blood-brain barrier as it could be important for the absorption of some drugs and their availability at the site of inflammation.

## 7. Treatment Strategies for Modulating the Microbiome in MS

The existing human studies of microbiome composition have limitations and have not yet identified clear patterns of altered gut microbiota in people with MS. Nonetheless, there is sufficient evidence for the role of microbiome changes in the pathophysiology of autoimmune diseases, including MS; so much so that it has been identified as a potential therapeutic target. The immunomodulatory properties of both antibiotics and probiotics and their presumed beneficial effects on autoimmune diseases were studied both in the experimental animal model (EAE) and MS.

### 7.1. Antibiotics

It was observed that a cocktail of broad-spectrum antibiotics impaired the development of EAE [43] and modified the outcome of the progressive stage of EAE [44]. In one human clinical trial, treatment with minocycline showed a decrease in the risk of conversion to MS, change in the lesion volume, and the absence of new enhancing lesions at six months of treatment, however, the effect did not last beyond 24 months of study.

### 7.2. Probiotics

Some studies report a positive effect of probiotics, whereas recent meta-analysis in EAE is not very encouraging [45]. Probiotics might have a role in regulating intestinal microbial homeostasis, maintaining the gastrointestinal barrier function, interfering with the ability of pathogens to colonize, and even the modulation of local and systemic immune responses. The meta-analysis concluded that the administration of probiotics is associated with a significant reduction in the risk of mortality, but only in female animals. Moreover, the meta-analysis revealed the promising effects of probiotics in the prevention and management of EAE (decreased incidence, delayed onset of symptoms, and milder symptoms). Most promising results were obtained while using Enterococci bacteria. The authors conclude that it is worth conducting trials in humans [45].

A recent meta-analysis in patients with relapsing-remitting MS found four trials with 213 patients (106 with intervention) and concluded that there was an improvement in disability, depression, and general health in patients using probiotics [46]. This should be taken with caution. These trials were relatively small, had a short duration, and did not provide sufficient information about the immunomodulatory treatments used, dietary habits, etc. to assess the activity of the disease. A study of nine MS patients showed some effect on microbiome composition and a shift towards anti-inflammatory cytokines in the blood during the weeks of treatment with probiotics [47]. 

### 7.3. Parasites

There exists a negative correlation between the geographical distribution of autoimmune diseases and helminth infections. Helminths may be important environmental regulators for tolerance induction against autoimmunity, in accordance with the original hygiene hypothesis proposed in 1989. Helminths regulate inflammation to their benefit, including strong Th2-biased responses, as well as the induction of many regulatory cells such as alternatively activated macrophages (AAMs), myeloid-derived suppressor cells (MDSCs), T-regulatory (Tregs), B-regulatory (Bregs), and tolerogenic dendritic cells (iDCs) [48]. It is hypothesized that helminths may have the potential ability to downregulate inflammatory diseases, which has been studied in the EAE model using several types of helminth infections including *T. crassiceps*, *S. mansoni*, *T. pseudospiralis*, *T. spiralis*, and *F. hepatica*. All these infections were shown to potently modulate EAE in association with a Th2-type response that is low in TNF-α, IFN-γ, and IL-17A. Some studies used helminth-derived products to mimic the effect of the whole infection with the same result. The potency of this treatment was superior even to that observed for dexamethasone [48].

In MS patients, the observation that autoimmune downregulation occurs secondary to parasite infections (fewer and milder relapses and less MRI activity) has been explained by the induction of regulatory T cells secreting suppressive cytokines IL-10 and TGF-b, as well as CD4þ, CD25þ, and FoxP3þ T cells displaying significant suppressive function [49]. Following anti-helminth drug administration, clinical and radiological activity increased to levels observed in uninfected MS patients. Mechanistic studies revealed that the cytokine secretion patterns reverted to their previous state [50]. An exploratory study in five MS patients at the University of Nottingham received 2500 nonpathogenic helminth *T. suis* ova every two weeks for three months. MRI enhancing lesion numbers fell around 70% at the end of the treatment but returned to baseline values two months after treatment discontinuation [51]. However, the use of live parasites is not optimal. The compliance of patients is predicted to be generally poor, and this is largely because of the negative attitudes associated with the concept of being infected with live pathogens. There may occur unexpected side effects and the production of the tested material in compliance with good manufacturing practice would be extremely difficult.

A safer and more reliable alternative to live infection would be to identify and use the specific immune-modulatory molecules produced by helminth parasites [52]. This would allow us to better understand the mechanism of action and dose the molecules to test the optimal efficacy of the treatment.

### 7.4. Fecal Microbiota Transplantation (FMT)

Case reports of the miraculous effects of FMT in MS patients can be found in the scientific literature. Fecal microbiota transplantation (FMT) appears to be an effective treatment for the *Clostridium difficile* infection and inflammatory bowel syndrome due to its ability to restore gut microbiota diversity [53]. One study found that after FMT treatment for constipation, three wheelchair-bound MS patients had such dramatic improvement in their neurological symptoms that they regained the ability to walk unassisted [54,55]. Rebuilding gut microbiota has been proposed as an innovative approach to MS treatment but it requires well-designed controlled studies.

In animal studies, FMT led to a reduced abundance of the Akkermansia genus (in phylum Verrucomicrobia) and an elevated abundance of the Prevotella genus (in phylum Bacteroidetes) in gut microbiota, which recalls the findings of decreased gut Akkermansia after probiotic intervention [56] and increased gut Prevotella after first-line disease-modifying treatment [57] and intermittent fasting in MS patients [58]. In EAE, FMT had a therapeutic effect delaying the onset of disease and reducing clinical severity [59]. 

A detailed study including metagenomics in a single MS patient after FMT showed not only an improved composition of gut microbiota with a high sustained production of SCFAs, improved gait, and no relapses during a year of follow up but also a sustained increase in the serum levels of brain-derived neurotrophic factor (BDNF), which is known to be low in MS. BDNF levels correlated with the production of SCFA in the gut. One possible mechanism for increased BDNF is an increased abundance of butyrate-producers after FMT, because BDNF-production is inhibited by the immune systemic inflammatory state and butyrate has anti-inflammatory activities [60]. Phase 1b and 2 studies are ongoing to assess the effect of FMT in MS [61]. 

## 8. Recommendations for Further Research

**Figure 2:**
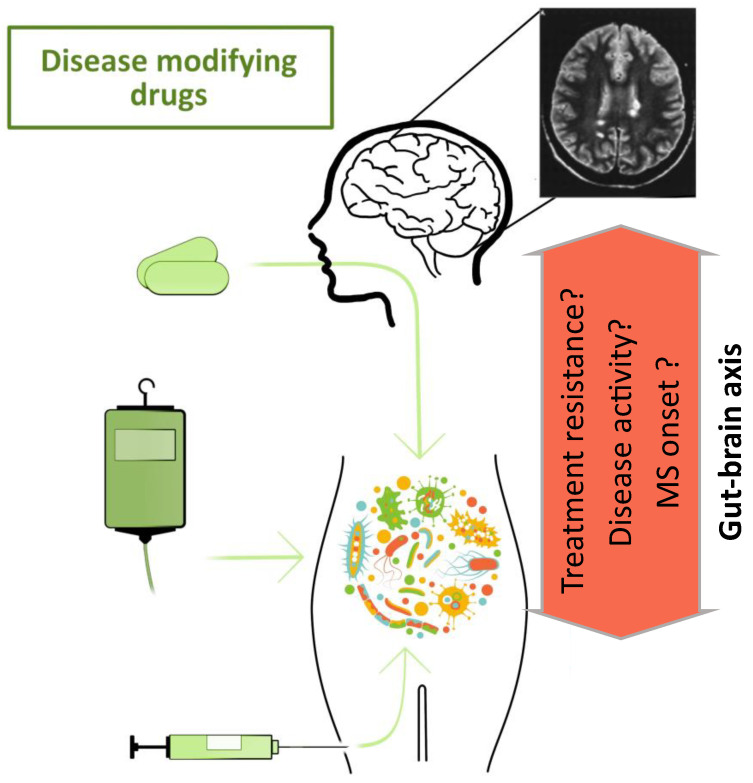
Schematics of the role of the gut-brain axis in the course and treatment of multiple sclerosis.

The crucial step in understanding the role of microbiota in the course of MS is to understand its role in the onset and the early stages of the disease and its changes throughout the treatment (Figure 2). 

The effect of corticotherapy commonly used to treat MS relapses on the microbiome has not been investigated in either the short or long term. Though there is some knowledge on the effect of first-line therapies (interferon beta and glatiramer acetate) on the microbiome of MS patients, the role of the microbiome in first-line treatment failure is still unknown. There is a lack of knowledge about the long-term effects of high efficacy therapeutic options (cladribine, ocrelizumab, and fingolimod) on the gut microbiota during the treatment process and how it correlates with treatment effectiveness; although some studies have been announced [62]. 

Studies in the field of gut microbiota may bring a new quality to the overall management of MS by either adding recommendations for MS patients in terms of treatment strategies or by influencing their microbiota completely in addition to increasing the armamentarium of drugs being developed to modulate the immune response in MS. Unfortunately, these drugs are not 100% effective in all patients, and research in new areas, such as the modulation of gut microbiota composition, is warranted.

## Data Availability

Not applicable.

## References

[1] Sadovnick A.D., Armstrong H., Rice G.P., Bulman D., Hashimoto L., Paty D.W., Hashimoto S.A., Warren S., Hader W., Murray T.J. (1993). A population-based study of multiple sclerosis in twins: Update. Ann. Neurol..

[2] Hollenbach J.A., Oksenberg J.R. (2015). The immunogenetics of multiple sclerosis: A comprehensive review. J. Autoimmun..

[3] Bjornevik K., Cortese M., Healy B.C., Kuhle J., Mina M.J., Leng Y., Elledge S.J., Niebuhr D.W., Scher A.I., Munger K.L. (2022). Longitudinal analysis reveals high prevalence of Epstein-Barr virus associated with multiple sclerosis. Science.

[4] Magliozzi R., Serafini B., Rosicarelli B., Chiappetta G., Veroni C., Reynolds R., Aloisi F. (2013). B-cell enrichment and Epstein-Barr virus infection in inflammatory cortical lesions in secondary progressive multiple sclerosis. J. Neuropathol. Exp. Neurol..

[5] Grigoriadis N., van Pesch V., Paradig M.S.G. (2015). A basic overview of multiple sclerosis immunopathology. Eur. J. Neurol..

[6] Human Microbiome Project Consortium (2012). Structure, function and diversity of the healthy human microbiome. Nature.

[7] Jumpstart Consortium Human Microbiome Project Data Generation Working Group (2012). Evaluation of 16S rDNA-based community profiling for human microbiome research. PLoS ONE.

[8] Qin J., Li R., Raes J., Arumugam M., Burgdorf K.S., Manichanh C., Nielsen T., Pons N., Levenez F., Yamada T. (2010). A human gut microbial gene catalogue established by metagenomic sequencing. Nature.

[9] Mowat A.M., Agace W.W. (2014). Regional specialization within the intestinal immune system. Nat. Rev. Immunol..

[10] Mueller S., Saunier K., Hanisch C., Norin E., Alm L., Midtvedt T., Cresci A., Silvi S., Orpianesi C., Verdenelli M.C. (2006). Differences in fecal microbiota in different European study populations in relation to age, gender, and country: A cross-sectional study. Appl. Environ. Microbiol..

[11] Johnson C.L., Versalovic J. (2012). The human microbiome and its potential importance to pediatrics. Pediatrics.

[12] Tlaskalova-Hogenova H., Stepankova R., Hudcovic T., Tuckova L., Cukrowska B., Lodinova-Zadnikova R., Kozakova H., Rossmann P., Bartova J., Sokol D. (2004). Commensal bacteria (normal microflora), mucosal immunity and chronic inflammatory and autoimmune diseases. Immunol. Lett..

[13] Belkaid Y., Hand T.W. (2014). Role of the microbiota in immunity and inflammation. Cell.

[14] Piovani D., Danese S., Peyrin-Biroulet L., Nikolopoulos G.K., Lytras T., Bonovas S. (2019). Environmental Risk Factors for Inflammatory Bowel Diseases: An Umbrella Review of Meta-analyses. Gastroenterology.

[15] Zarate-Blades C.R., Horai R., Caspi R.R. (2016). Regulation of Autoimmunity by the Microbiome. DNA Cell Biol..

[16] Round J.L., Mazmanian S.K. (2009). The gut microbiota shapes intestinal immune responses during health and disease. Nat. Rev. Immunol..

[17] Palm N.W., de Zoete M.R., Flavell R.A. (2015). Immune-microbiota interactions in health and disease. Clin. Immunol..

[18] Hrncir T., Stepankova R., Kozakova H., Hudcovic T., Tlaskalova-Hogenova H. (2008). Gut microbiota and lipopolysaccharide content of the diet influence development of regulatory T cells: Studies in germ-free mice. BMC Immunol..

[19] Kim M., Kim C.H. (2017). Regulation of humoral immunity by gut microbial products. Gut Microbes.

[20] Freedman S.N., Shahi S.K., Mangalam A.K. (2018). The “Gut Feeling”: Breaking Down the Role of Gut Microbiome in Multiple Sclerosis. Neurotherapeutics.

[21] Thompson A.J., Banwell B.L., Barkhof F., Carroll W.M., Coetzee T., Comi G., Correale J., Fazekas F., Filippi M., Freedman M.S. (2018). Diagnosis of multiple sclerosis: 2017 revisions of the McDonald criteria. Lancet Neurol..

[22] Laaker C., Hsu M., Fabry Z., Miller S.D., Karpus W.J. (2021). Experimental Autoimmune Encephalomyelitis in the Mouse. Curr. Protoc..

[23] Westall F.C. (2006). Molecular mimicry or structural mimicry?. Mol. Immunol..

[24] Kuhlmann T., Ludwin S., Prat A., Antel J., Bruck W., Lassmann H. (2017). An updated histological classification system for multiple sclerosis lesions. Acta Neuropathol..

[25] Srpova B., Uher T., Hrnciarova T., Barro C., Andelova M., Michalak Z., Vaneckova M., Krasensky J., Noskova L., Havrdova E.K. (2021). Serum neurofilament light chain reflects inflammation-driven neurodegeneration and predicts delayed brain volume loss in early stage of multiple sclerosis. Mult. Scler..

[26] Bittner S., Oh J., Havrdova E.K., Tintore M., Zipp F. (2021). The potential of serum neurofilament as biomarker for multiple sclerosis. Brain.

[27] Bar-Or A., Pender M.P., Khanna R., Steinman L., Hartung H.P., Maniar T., Croze E., Aftab B.T., Giovannoni G., Joshi M.A. (2020). Epstein-Barr Virus in Multiple Sclerosis: Theory and Emerging Immunotherapies. Trends Mol. Med..

[28] Thorley-Lawson D.A. (2001). Epstein-Barr virus: Exploiting the immune system. Nat. Rev. Immunol..

[29] Corcione A., Aloisi F., Serafini B., Capello E., Mancardi G.L., Pistoia V., Uccelli A. (2005). B-cell differentiation in the CNS of patients with multiple sclerosis. Autoimmun. Rev..

[30] Wekerle H. (2017). Brain Autoimmunity and Intestinal Microbiota: 100 Trillion Game Changers. Trends Immunol..

[31] Berer K., Gerdes L.A., Cekanaviciute E., Jia X., Xiao L., Xia Z., Liu C., Klotz L., Stauffer U., Baranzini S.E. (2017). Gut microbiota from multiple sclerosis patients enables spontaneous autoimmune encephalomyelitis in mice. Proc. Natl. Acad. Sci. USA.

[32] Atarashi K., Tanoue T., Shima T., Imaoka A., Kuwahara T., Momose Y., Cheng G., Yamasaki S., Saito T., Ohba Y. (2011). Induction of colonic regulatory T cells by indigenous Clostridium species. Science.

[33] Wang Y., Yin Y., Chen X., Zhao Y., Wu Y., Li Y., Wang X., Chen H., Xiang C. (2019). Induction of Intestinal Th17 Cells by Flagellins From Segmented Filamentous Bacteria. Front. Immunol..

[34] Berer K., Mues M., Koutrolos M., Rasbi Z.A., Boziki M., Johner C., Wekerle H., Krishnamoorthy G. (2011). Commensal microbiota and myelin autoantigen cooperate to trigger autoimmune demyelination. Nature.

[35] Cekanaviciute E., Yoo B.B., Runia T.F., Debelius J.W., Singh S., Nelson C.A., Kanner R., Bencosme Y., Lee Y.K., Hauser S.L. (2017). Gut bacteria from multiple sclerosis patients modulate human T cells and exacerbate symptoms in mouse models. Proc. Natl. Acad. Sci. USA.

[36] Probstel A.K., Baranzini S.E. (2018). The Role of the Gut Microbiome in Multiple Sclerosis Risk and Progression: Towards Characterization of the “MS Microbiome”. NeuroTherapeutics.

[37] Bach J.F. (2020). Revisiting the Hygiene Hypothesis in the Context of Autoimmunity. Front. Immunol..

[38] Vatanen T., Kostic A.D., d’Hennezel E., Siljander H., Franzosa E.A., Yassour M., Kolde R., Vlamakis H., Arthur T.D., Hamalainen A.M. (2016). Variation in Microbiome LPS Immunogenicity Contributes to Autoimmunity in Humans. Cell.

[39] Wasko N.J., Nichols F., Clark R.B. (2020). Multiple sclerosis, the microbiome, TLR2, and the hygiene hypothesis. Autoimmun. Rev..

[40] Yacyshyn B., Meddings J., Sadowski D., Bowen-Yacyshyn M.B. (1996). Multiple sclerosis patients have peripheral blood CD45RO+ B cells and increased intestinal permeability. Dig. Dis. Sci..

[41] Nouri M., Bredberg A., Westrom B., Lavasani S. (2014). Intestinal barrier dysfunction develops at the onset of experimental autoimmune encephalomyelitis, and can be induced by adoptive transfer of auto-reactive T cells. PLoS ONE.

[42] Buscarinu M.C., Romano S., Mechelli R., Pizzolato Umeton R., Ferraldeschi M., Fornasiero A., Renie R., Cerasoli B., Morena E., Romano C. (2018). Intestinal Permeability in Relapsing-Remitting Multiple Sclerosis. Neurotherapeutics.

[43] Ochoa-Reparaz J., Mielcarz D.W., Ditrio L.E., Burroughs A.R., Foureau D.M., Haque-Begum S., Kasper L.H. (2009). Role of gut commensal microflora in the development of experimental autoimmune encephalomyelitis. J. Immunol..

[44] Colpitts S.L., Kasper E.J., Keever A., Liljenberg C., Kirby T., Magori K., Kasper L.H., Ochoa-Reparaz J. (2017). A bidirectional association between the gut microbiota and CNS disease in a biphasic murine model of multiple sclerosis. Gut Microbes.

[45] Valizadeh S., Majdi Seghinsara A., Maleki Chollou K., Bahadori A., Abbaszadeh S., Taghdir M., Behniafar H., Riahi S.M. (2021). The efficacy of probiotics in experimental autoimmune encephalomyelitis (an animal model for MS): A systematic review and meta-analysis. Lett. Appl. Microbiol..

[46] Mirashrafi S., Hejazi Taghanaki S.Z., Sarlak F., Moravejolahkami A.R., Hojjati Kermani M.A., Haratian M. (2021). Effect of probiotics supplementation on disease progression, depression, general health, and anthropometric measurements in relapsing-remitting multiple sclerosis patients: A systematic review and meta-analysis of clinical trials. Int. J. Clin. Pract..

[47] Tankou S.K., Regev K., Healy B.C., Cox L.M., Tjon E., Kivisakk P., Vanande I.P., Cook S., Gandhi R., Glanz B. (2018). Investigation of probiotics in multiple sclerosis. Mult. Scler..

[48] Peon A.N., Ledesma-Soto Y., Olguin J.E., Bautista-Donis M., Sciutto E., Terrazas L.I. (2017). Helminth Products Potently Modulate Experimental Autoimmune Encephalomyelitis by Downregulating Neuroinflammation and Promoting a Suppressive Microenvironment. Mediat. Inflamm..

[49] Correale J., Farez M. (2007). Association between parasite infection and immune responses in multiple sclerosis. Ann. Neurol..

[50] Correale J., Farez M.F. (2011). The impact of parasite infections on the course of multiple sclerosis. J. Neuroimmunol..

[51] Fleming J.O., Isaak A., Lee J.E., Luzzio C.C., Carrithers M.D., Cook T.D., Field A.S., Boland J., Fabry Z. (2011). Probiotic helminth administration in relapsing-remitting multiple sclerosis: A phase 1 study. Mult. Scler..

[52] Dixit A., Tanaka A., Greer J.M., Donnelly S. (2017). Novel Therapeutics for Multiple Sclerosis Designed by Parasitic Worms. Int. J. Mol. Sci..

[53] Allegretti J.R., Mullish B.H., Kelly C., Fischer M. (2019). The evolution of the use of faecal microbiota transplantation and emerging therapeutic indications. Lancet.

[54] Borody T.J., Brandt L.J., Paramsothy S. (2014). Therapeutic faecal microbiota transplantation: Current status and future developments. Curr. Opin. Gastroenterol..

[55] Vendrik K.E.W., Ooijevaar R.E., de Jong P.R.C., Laman J.D., van Oosten B.W., van Hilten J.J., Ducarmon Q.R., Keller J.J., Kuijper E.J., Contarino M.F. (2020). Fecal Microbiota Transplantation in Neurological Disorders. Front. Cell Infect. Microbiol..

[56] Tankou S.K., Regev K., Healy B.C., Tjon E., Laghi L., Cox L.M., Kivisakk P., Pierre I.V., Hrishikesh L., Gandhi R. (2018). A probiotic modulates the microbiome and immunity in multiple sclerosis. Ann. Neurol..

[57] Jangi S., Gandhi R., Cox L.M., Li N., von Glehn F., Yan R., Patel B., Mazzola M.A., Liu S., Glanz B.L. (2016). Alterations of the human gut microbiome in multiple sclerosis. Nat. Commun..

[58] Cignarella F., Cantoni C., Ghezzi L., Salter A., Dorsett Y., Chen L., Phillips D., Weinstock G.M., Fontana L., Cross A.H. (2018). Intermittent Fasting Confers Protection in CNS Autoimmunity by Altering the Gut Microbiota. Cell Metab..

[59] Li K., Wei S., Hu L., Yin X., Mai Y., Jiang C., Peng X., Cao X., Huang Z., Zhou H. (2020). Protection of Fecal Microbiota Transplantation in a Mouse Model of Multiple Sclerosis. Mediat. Inflamm..

[60] Engen P.A., Zaferiou A., Rasmussen H., Naqib A., Green S.J., Fogg L.F., Forsyth C.B., Raeisi S., Hamaker B., Keshavarzian A. (2020). Single-Arm, Non-randomized, Time Series, Single-Subject Study of Fecal Microbiota Transplantation in Multiple Sclerosis. Front. Neurol..

[61] Schepici G., Silvestro S., Bramanti P., Mazzon E. (2019). The Gut Microbiota in Multiple Sclerosis: An Overview of Clinical Trials. Cell Transplant..

[62] Van Pamelen J., van Olst L., Budding A.E., Group B.I.A.S., de Vries H.E., Visser L.H. (2020). Alterations of Gut Microbiota and the Brain-Immune-Intestine Axis in Patients with Relapsing-Remitting Multiple Sclerosis After Treatment With Oral Cladribine: Protocol for a Prospective Observational Study. JMIR Res. Protoc..

